# Knowledge, perceptions, and usage patterns of nicotine pouches among Saudi medical students: A cross-sectional study

**DOI:** 10.18332/tid/207914

**Published:** 2025-09-08

**Authors:** Najim Z. Alshahrani

**Affiliations:** 1 Department of Family and Community Medicine, Faculty of Medicine, University of Jeddah, Jeddah, Saudi Arabia

**Keywords:** nicotine pouches, smoking cessation, medical students, public health, Saudi Arabia

## Abstract

**INTRODUCTION:**

Tobacco smoking continues to pose a major global public health challenge. Medical students play a crucial role in shaping future smoking cessation practices. Nicotine pouches have recently emerged as a tobacco-free alternative with a potentially reduced harm profile. However, little is known about their use and perception among medical students in Saudi Arabia. This study aimed to assess medical students’ knowledge and perceptions of nicotine pouches and to examine usage patterns among those who consume these products.

**METHODS:**

A cross-sectional study was conducted between April and July 2024 among 295 medical students from three universities in Saudi Arabia. Data were collected through a self-administered electronic questionnaire covering demographics, smoking history, knowledge, perceptions, and usage behaviors. Statistical analyses, including chi-squared tests and logistic regression, were used to identify factors associated with knowledge and usage.

**RESULTS:**

Smoking prevalence among participants was 16.3%, with significantly higher rates among males. Overall, 58.6% of students demonstrated good knowledge of nicotine pouches. Higher knowledge scores were associated with male gender, senior academic year, and higher grade point average (GPA). Among users, 62.9% reported quitting smoking, and more than half noted health improvements. The 10 mg nicotine strength was the most commonly used. Social influence, particularly peer pressure, was the primary reason for use. Despite noting harm reduction potential, students expressed concern about nicotine dependence and the need for stronger regulation.

**CONCLUSIONS:**

Saudi medical students show moderate knowledge of nicotine pouches, influenced by academic and demographic factors. However, concerns about dependence and regulation highlight the need for targeted education and policy development. Integrating this topic into medical curricula may better prepare future physicians to address nicotine use in clinical practice.

## INTRODUCTION

Smoking continues to be a major global public health concern, contributing significantly to morbidity, mortality, and economic burden worldwide^1^. It is a well-established risk factor for a wide range of diseases, including cancers of the lung, upper respiratory tract, gastrointestinal system, kidneys, pancreas, liver, bladder, and cervix, as well as cardiovascular disease, stroke, and chronic obstructive pulmonary disease (COPD), including emphysema and chronic bronchitis^2,3^. Additionally, tobacco use has been associated with increased vulnerability to tuberculosis, ocular disorders, autoimmune conditions such as rheumatoid arthritis, and impaired immune function^4,5^. The financial impact of smoking is substantial; in 2012, tobacco use accounted for 5.6% of global health expenditures, with total economic costs amounting to 1.8% of the global GDP^6^.

The majority of smokers initiate tobacco use during young adulthood, particularly between the ages of 18 and 25 years^7^. During this period, individuals are especially susceptible to external influences such as peer pressure, stress, and social norms^8^. Initiating smoking at a younger age is associated with a higher likelihood of long-term nicotine dependence and reduced success in quitting^9^. According to a national study conducted in the United States, 43.4% of adults had attempted to quit smoking for at least one day within the past year, but only 9.1% achieved sustained abstinence, illustrating the difficulty of cessation efforts^10^.

In Saudi Arabia, smoking among university students remains a public health issue. A 2019 meta-analysis reported an overall smoking prevalence of 17% among Saudi college students, with a higher rate among males (26%) compared to females (5%)^11^. A study conducted at Jazan University found a 12.4% smoking rate among medical students, with waterpipe use particularly common, reported by 47% of male and 77.8% of female smokers^12^. Academic performance has also been found to correlate with smoking behavior; students with higher GPAs were less likely to smoke^12^.

Nicotine pouches are a relatively new form of oral nicotine delivery that has gained visibility in several countries, including Saudi Arabia. Unlike traditional smokeless tobacco products such as toombak or shammah, nicotine pouches are free of tobacco leaf and instead contain pharmaceutical-grade nicotine, flavorings, and an inert cellulose base^13^. These pouches are placed between the gum and lip, allowing nicotine to be absorbed through the oral mucosa. Products currently available in the Saudi market, such as DZRT, are offered in various dosages (3, 6, and 10 mg) and flavored varieties^13^. Although these products do not require combustion or spitting and are marketed for their convenience and cleanliness, there are growing concerns about their potential to cause nicotine dependence, especially among young adults^14^.

This study examines the level of awareness, knowledge, and perceptions regarding nicotine pouches among medical students in Saudi Arabia. In addition, for participants who reported using nicotine pouches, the study explores usage patterns, including dosage preferences and reasons for use. Understanding these factors among medical students may help inform future educational efforts and research related to nicotine product use within this population.

## METHODS

### Study design and study period

A descriptive cross-sectional study with an analytic component was conducted between April and July 2024 in Jeddah, Saudi Arabia. The study targeted medical students from three universities: the University of Jeddah, King Abdulaziz University, and Batterjee Medical College. This study was reviewed and approved by the Bioethics Committee of Scientific and Medical Research at the University of Jeddah. Ethical approval was granted under Application Number (UJ-REC-225) and Bioethics Committee Registration Number (HAP-02-J-094).

### Sample size

The sample size was calculated using OpenEpi online calculator, focusing on the prevalence of current smoking among medical students as the primary outcome. Based on a previous study conducted in Jeddah among medical students^14^, which reported a current smoking prevalence of 21.6%, the sample size was determined with a 95% confidence level and a 5% margin of error (alpha=0.05), resulting in a minimum required sample size of 254 participants. Data collection continued until the target sample size was reached. To enhance statistical power, we recruited additional participants, resulting in a final sample size of 295.

### Sampling and data collection approach

Convenience sampling was employed to recruit participants. Data collection commenced on 1 April 2024 and continued until the required sample size was reached. A self-administered, structured questionnaire was distributed electronically using Google Forms. The survey link was disseminated through official academic communication channels and student representatives at each participating university. To expand outreach while maintaining target population integrity, the link was also shared on closed student groups on Telegram and WhatsApp that required university email verification or administrator approval for access. To prevent unauthorized participation, the questionnaire included an eligibility question confirming medical student status, and responses lacking institutional affiliation or containing duplicate IP addresses were reviewed and excluded where necessary. All participation was voluntary, and responses were collected anonymously.

### Study tools

The survey instrument was structured into four distinct sections to capture specific aspects of the study objectives. The first section collected data on participants’ demographic profiles, including age, gender, university affiliation, academic year, grade point average (GPA), and smoking history. The second section focused specifically on participants who reported using nicotine pouches, exploring factors such as the duration of use, reasons for adoption, preferred nicotine dosage levels (3, 6, or 10 mg), usage patterns, and perceived effects of nicotine pouches on smoking behavior. The third section evaluated participants’ knowledge about nicotine pouches through a set of 10 true/false questions designed to assess the understanding of key concepts related to nicotine pouches and their role in harm reduction. Responses were coded as 1 for correct answers and 0 for incorrect answers, and knowledge levels were categorized as ‘good’ or ‘poor’ based on a median split, with scores of 6 to 10 classified as good knowledge and scores of 0 to 5 as poor knowledge. The fourth section assessed participants’ perceptions of nicotine pouches using 10 items rated on a 5-point Likert scale (1=strongly disagree, 5=strongly agree), with higher scores indicating more favorable perceptions of nicotine pouches and their potential role in reducing smoking-related harm.

### Development and validation of the tool

The questionnaire was developed based on an extensive literature review to ensure alignment with existing research on nicotine pouches^15-18^. Following initial development, the survey instrument underwent review by four experts specializing in public health to assess its content validity and clarity. Their recommendations were incorporated to refine the questions. A pilot study was conducted with 30 medical students to pre-test the instrument for readability, comprehension, and functionality. Feedback from the pilot participants resulted in minor revisions to improve clarity and reduce ambiguity. Internal consistency of the questionnaire was assessed using Cronbach’s alpha reliability coefficient, which demonstrated moderate to good reliability (Cronbach’s α=0.79). In addition, face validity was ensured by seeking feedback on the questionnaire’s relevance and clarity from the target population during the pilot phase.

### Statistical analysis

Data collected from the survey were analyzed using the Statistical Package for the Social Sciences (SPSS, version 26). Descriptive statistics were used to summarize demographic characteristics, smoking behavior, knowledge levels, perceptions, and nicotine pouch usage patterns. Categorical variables were expressed as frequencies and percentages, while continuous variables were summarized as means and standard deviations. Associations between demographic and behavioral variables and the study outcomes were evaluated using chi-squared tests for categorical variables and independent t-tests for continuous variables. Binary logistic regression was employed to identify factors associated with good knowledge regarding nicotine pouches. Adjusted odds ratios (AORs) with 95% confidence intervals (CIs) were calculated to assess the strength of associations between knowledge levels and factors such as university affiliation, academic year, GPA, and awareness of nicotine pouches. Multivariable regression analysis was used to control potential confounders. For perception-related data, Likert scale responses were analyzed using medians and interquartile ranges (IQRs). Group comparisons were conducted using Mann-Whitney U tests to identify significant differences between smokers and non-smokers. All statistical tests were two-tailed. A p<0.05 was considered statistically significant for all analyses.

## RESULTS

### Demographics of study participants by smoking status

The study involved 295 medical students, with 48 (16.3%) identified as current smokers and 247 (83.7%) as non-smokers (Table 1). Statistically significant differences were observed in several demographic variables. Current smokers were slightly older (23.4 ± 1.7 years) than non-smokers (22.3 ± 2.2 years; p<0.01). Gender distribution differed significantly, with males comprising a higher percentage of current smokers (85.4%) compared to non-smokers (53.8%; p<0.01). The majority of participants were from the University of Jeddah; however, the differences in university affiliation were not statistically significant (p=0.07). The academic year also showed notable variations, with a greater proportion of current smokers in their fifth year (37.5%) compared to non-smokers, who were more evenly distributed across years (p<0.01). Academic performance, as measured by GPA, also differed significantly between groups. Current smokers were more likely to have a GPA between 3.51 and 4.50 (66.7%), while non-smokers more frequently had a GPA >4.5 (44.9%; p=0.02). Notably, current smokers were significantly more likely to have used nicotine pouches (27.1%) compared to non-smokers (11.3% and 8.9%, respectively; p<0.01).

**Table 1:** Demographic characteristics of Saudi medical students by smoking status, a cross-sectional study, Saudi Arabia, April–July 2024 (N=295)

*Characteristics*	*Total* *(N=295)* *n (%)*	*Current smokers* *(N=48)* *n (%)*	*Non-smokers* *(N=247)* *n (%)*	*p*
**Age** (years), mean ± SD	22.5 ± 2.1	23.4 ± 1.7	22.3 ± 2.2	<0.01*
**Gender**				<0.01*
Male	174 (59.0)	41 (85.4)	133 (53.8)	
Female	121 (41.0)	7 (14.6)	114 (46.2)	
**University**				0.07
University of Jeddah	179 (60.7)	36 (75.0)	143 (57.9)	
King Abdulaziz University	68 (23.1)	8 (16.7)	60 (24.3)	
Batterjee Medical College	48 (16.3)	4 (8.3)	44 (17.8)	
**Year of study**				<0.01*
First	24 (8.1)	0 (0.0)	24 (9.7)	
Second	31 (10.5)	6 (12.5)	25 (10.1)	
Third	34 (11.5)	5 (10.4)	29 (11.7)	
Fourth	85 (28.8)	13 (27.1)	72 (29.1)	
Fifth	51 (17.3)	18 (37.5)	33 (13.4)	
Sixth	37 (12.5)	5 (10.4)	32 (13.0)	
Internship	33 (11.2)	1 (2.1)	32 (13.0)	
**Academic GPA**				0.02*
<2.50	1 (0.3)	0 (0.0)	1 (0.4)	
2.50–3.50	34 (11.5)	3 (6.3)	31 (12.6)	
3.51–4.50	136 (46.1)	32 (66.7)	104 (42.1)	
>4.5	124 (42.0)	13 (27.1)	111 (44.9)	
**Heard about nicotine pouches**				<0.01*
No	137 (46.4)	5 (10.4)	132 (53.4)	
Yes	158 (53.6)	43 (89.6)	115 (46.6)	
**Used nicotine pouches**				<0.01*
No	260 (88.1)	35 (72.9)	225 (91.1)	
Yes	35 (11.9)	13 (27.1)	22 (8.9)	

GPA: grade point average. P-values calculated using independent samples t-test for age and chi-squared test for categorical variables.

* Significant values at p<0.05.

### Knowledge about nicotine pouches

Overall, 58.6% of participants were categorized as having good knowledge about nicotine pouches, while 41.4% had poor knowledge. Knowledge levels about nicotine pouches showed significant differences across demographic and behavioral variables (Table 2). Participants with good knowledge had a higher mean age (22.8 ± 1.9 years) compared to those with poor knowledge (22.0 ± 2.3 years; p<0.01). Gender played a role, with males more likely to demonstrate good knowledge (62.6%) compared to females (52.9%; p<0.01). University affiliation also influenced knowledge levels, with students from King Abdulaziz University showing the highest proportion of good knowledge (72.1%), followed by the University of Jeddah (58.7%) and Batterjee Medical College (39.6%; p<0.01). The academic year was another significant factor, with higher proportions of good knowledge observed in the fifth (66.7%) and fourth years (65.9%) compared to the second year (40.0%; p=0.02). Academic GPA correlated positively with knowledge, as students with a GPA >4.5 had the highest proportion of good knowledge (63.7%; p=0.04). Smoking status showed a trend, with current smokers demonstrating better knowledge (70.8%) than non-smokers (56.3%), though this difference was not statistically significant (p=0.06). Usage of nicotine pouches did not show significant associations with knowledge levels (p=0.6 and p=0.2, respectively).

**Table 2:** Knowledge levels about nicotine pouches among Saudi medical students, a cross-sectional study, Saudi Arabia, April–July 2024 (N=295)

*Characteristics*	*Categories*	*Good knowledge* *n (row %)*	*Poor knowledge* *n (row %)*	*p*
**Age** (years), mean ± SD		22.8 ± 1.9	22.0 ± 2.3	<0.01*
**Gender**	Female	64 (52.9)	57 (47.1)	<0.01*
	Male	109 (62.6)	65 (37.4)	
**University**	University of Jeddah	105 (58.7)	74 (41.3)	<0.01*
	King Abdulaziz University	49 (72.1)	19 (27.9)	
	Batterjee Medical College	19 (39.6)	29 (60.4)	
**Year of study**	Second	22 (40.0)	33 (60.0)	0.02*
	Third	19 (55.9)	15 (44.1)	
	Fourth	56 (65.9)	29 (34.1)	
	Fifth	34 (66.7)	17 (33.3)	
	Sixth	42 (60.0)	28 (40.0)	
**Academic GPA**	<3.50	14 (40.0)	21 (60.0)	0.04*
	3.51–4.50	80 (58.8)	56 (41.2)	
	>4.5	79 (63.7)	45 (36.3)	
**Smoking status**	Current smoker	34 (70.8)	14 (29.2)	0.06
	Non-smoker	139 (56.3)	108 (43.7)	
**Used nicotine pouches before**	No	149 (57.3)	111 (42.7)	0.2
	Yes	24 (68.6)	11 (31.4)	
**Overall**		173 (58.6)	122 (41.4)	

GPA: grade point average. P-values calculated using independent samples t-test for age and chi-squared test for categorical variables.

* Significant values at p<0.05.

### Perceptions towards nicotine pouches

Participants’ perceptions of nicotine pouches were assessed using a Likert scale, with responses coded from 1 (strongly disagree) to 5 (strongly agree) (Table 3). Both current smokers and non-smokers agreed on the potential benefits of nicotine pouches in reducing the public health burden associated with smoking, with a median score of 4 for both groups (p=0.3). Participants also expressed strong agreement that medical students should receive formal education about the use and effects of nicotine pouches as part of their curriculum, though non-smokers showed slightly higher agreement (median: 4) compared to current smokers (median: 3.5; p=0.3). Concerns about nicotine pouches potentially re-normalizing smoking behaviors in society were more pronounced among non-smokers (median: 4) than current smokers (median: 3), though this difference was not statistically significant (p=0.1). Similarly, non-smokers were more likely to believe that the benefits of nicotine pouches in smoking cessation outweigh their potential risks (median: 4) compared to current smokers (median: 3.5; p=0.2). A significant difference was observed in comfort levels with discussing and recommending nicotine pouches, where nonsmokers expressed greater comfort (median: 4) than current smokers (median: 3; p=0.04). Participants across both groups strongly agreed on the need for stronger regulatory frameworks for nicotine pouches to ensure their safety and efficacy (median: 4; p=0.7). Additionally, both groups expressed skepticism about the involvement of the tobacco industry in the development and marketing of nicotine pouches (median: 3; p=0.7) and concern about non-smokers potentially starting nicotine use through nicotine pouches, increasing overall nicotine dependence (median: 3; p=0.3). Finally, participants emphasized the importance of healthcare professionals staying informed about the latest research on nicotine pouches to provide evidence-based recommendations, with similar median scores for both groups (median: 4; p=0.4).

**Table 3:** Perceptions^a^ of nicotine pouches among Saudi medical students, stratified by smoking status, a crosssectional study, Saudi Arabia, April–July 2024 (N=295)

*Items*	*Current smokers* *Median (IQR)*	*Non-smokers* *Median (IQR)*	*p*
I believe that nicotine pouches can be a valuable tool in reducing the public health burden associated with smoking.	4 (2)	4 (3)	0.3
Medical students should receive formal education about the use and effects of nicotine pouches as part of their curriculum.	3.5 (3)	4 (4)	0.3
I am concerned that nicotine pouches might re-normalize smoking behaviors in society.	3 (3)	4 (4)	0.1
The benefits of nicotine pouches in smoking cessation outweigh their potential risks.	3.5 (2)	4 (4)	0.2
I would feel comfortable discussing and recommending nicotine pouches to patients as smoking cessation aids.	3 (4)	4 (4)	0.04*
Stronger regulatory frameworks for nicotine pouches are needed to ensure their safety and efficacy.	4 (3)	4 (4)	0.7
I am skeptical about the involvement of the tobacco industry in the development and marketing of nicotine pouches.	3 (3)	3 (4)	0.7
Public health campaigns should include information about nicotine pouches as part of comprehensive smoking cessation strategies.	4 (3)	4 (4)	0.8
I believe that non-smokers might start using nicotine through nicotine pouches, increasing overall nicotine dependence.	3 (3)	3 (4)	0.3
Healthcare professionals have a responsibility to stay informed about the latest research on nicotine pouches to provide evidence-based recommendations.	4 (2)	4 (4)	0.4

a Participants’ perceptions of nicotine pouches were assessed using a Likert scale, with responses coded from 1 (strongly disagree) to 5 (strongly agree). P-values calculated using Mann–Whitney U test.

* Significant values at p<0.05.

IQR: interquartile range.

### Factors associated with good knowledge regarding nicotine pouches

Logistic regression analysis identified several factors significantly associated with good knowledge about nicotine pouches (Table 4). University affiliation was a significant factor; students from King Abdulaziz University had higher odds of reporting good knowledge compared to those from the University of Jeddah (AOR=2.02; 95% CI: 1.02–3.90, p=0.04). The academic year also demonstrated a significant association. Fourth-year students had higher odds of good knowledge compared to second-year students (AOR=2.46; 95% CI: 1.18–5.13, p=0.02), as did fifth-year students (AOR=2.53; 95% CI: 1.10–5.78, p=0.03). Academic performance showed a significant relationship; students with a GPA >4.5 were more likely to have good knowledge (AOR=2.47; 95% CI: 1.06–5.72, p=0.04). Gender was not significantly associated with knowledge in either the univariate (OR=1.4; 95% CI: 0.9–2.3, p=0.09) or multivariate models (AOR=1.47; 95% CI: 0.84–2.57, p=0.10). Similarly, prior awareness of nicotine pouches did not show a statistically significant association in the adjusted model (p=0.50).

**Table 4:** Logistic regression analysis of factors associated with good knowledge about nicotine pouches among Saudi medical students, a cross-sectional study, Saudi Arabia, April–July 2024 (N=295)

*Variables*	*Univariate regression*		*Multivariate regression*	
	*OR (95% CI)*	*p*	*AOR (95% CI)*	*p*
**Gender**				
Female ^®^	1		1	
Male	1.4 (0.9–2.3)	0.09	1.47 (0.84–2.57)	0.1
**University**				
University of Jeddah ^®^	1	<0.01*	1	0.02*
King Abdulaziz University	1.8 (0.9–3.3)	0.05*	2.02 (1.02–3.9)	0.04*
Batterjee Medical College	0.4 (0.2–0.88)	0.02	0.61 (0.3–1.24)	0.17
**Year of study**				
Second ^®^	1	0.03*	1	0.14
Third	1.9 (0.8–4.5)	0.14	1.89 (0.76–4.6)	0.17
Fourth	2.9 (1.43–5.8)	<0.01*	2.46 (1.18–5.13)	0.02*
Fifth	3 (1.35–6.6)	<0.01*	2.53 (1.1–5.78)	0.03*
Sixth	2.2 (1.09–4.6)	0.03*	2.01 (0.9–4.45)	0.09
**Academic GPA**				
<3.50 ^®^	1	0.04*	1	0.1
3.51–4.50	2.14 (1.01–4.57)	0.04*	1.85 (0.8–4.24)	0.15
>4.5	2.63 (1.2–5.68)	0.01*	2.47 (1.06–5.72)	0.04*

GPA: grade point average. AOR: adjusted odds ratio; adjusted for university affiliation, year of study, and GPA.

* Significant values at p<0.05.

^®^ Reference categories.

### Nicotine pouch usage patterns and influencing factors

The distribution of nicotine dosage preferences among nicotine pouch users showed that 54.3% preferred a 10 mg dosage, followed by 37.1% who chose 6 mg, and 8.6% who opted for 3 mg (Supplementary file Figure 1). Patterns of nicotine dosage usage revealed that 80% of users maintained consistent nicotine levels over time, while 10% increased and another 10% decreased their dosage (Supplementary file Figure 2). Factors influencing nicotine pouch adoption were predominantly social, with 70% citing promotion by friends as the main reason, followed by 20% influenced by social media. Personal exploration and recommendations from bloggers accounted for 7% and 3%, respectively (Supplementary file Figure 3).

### Impact of nicotine pouch use on smoking behavior and health outcomes

The Sankey diagram (Supplementary file Figure 4) highlights the impact of nicotine pouch use among users. Of the 35 users, 62.9% (22 participants) reported that nicotine pouches helped them quit smoking cigarettes. Additionally, 51.4% (18 participants) noted improvements in overall health, and 45.7% (16 participants) reported enhanced stamina and physical health. A further 37.1% (13 participants) stated that nicotine pouches provided greater nicotine satisfaction compared to traditional cigarettes. A smaller group (17.1%, or 6 participants) cited ‘other’ impacts.

## DISCUSSION

This study examined smoking prevalence, awareness, perceptions, and use of nicotine pouches among Saudi medical students. The findings indicate that 16.3% of participants were current smokers, with smoking behavior associated with lower academic performance and more common among males. More than half of the respondents demonstrated adequate knowledge of nicotine pouches, with notable differences based on academic year and GPA. Among users of nicotine pouches, 62.9% reported having stopped smoking cigarettes. However, this should be interpreted cautiously due to the self-reported nature of the data and the use of a convenience sample.

The smoking prevalence observed in this sample closely mirrors national data on Saudi university students (17%) but exceeds the 12.4% reported in a prior study at Jazan University^11,12^. The variation may be influenced by regional differences, university culture, and access to tobacco products in metropolitan areas^19^. The higher prevalence among male students aligns with gender norms and social expectations in Saudi Arabia, where smoking among females is less socially acceptable^20^. Compared to global figures, smoking prevalence among medical students varies widely: 11% in the United Kingdom, 16.8% in the United States, and 23.4% in Egypt^21-23^. The association between smoking and lower GPA in our sample also reflects prior findings, where stress, academic pressure, and lifestyle factors contribute to tobacco use among students^12^.

Knowledge about nicotine pouches was significantly higher among male students, those in senior academic years, and students with higher GPAs. This trend is consistent with prior findings from Jazan and Riyadh, where greater academic exposure and curriculum content enhanced awareness of smoking-related topics^12,24^ . By contrast, a 2014 multi-institutional study across three Saudi medical schools found that 91.4% of students lacked sufficient knowledge about tobacco use, with an average score of 53% on related assessments^25^. While direct comparisons are limited, improvements in medical education, public health campaigns, and policy awareness may help explain the better knowledge scores in our sample.

Earlier international research also documented substantial gaps in medical students’ understanding of tobacco use and cessation tools. For example, Polish medical students showed both a high prevalence of smoking and limited understanding of its health consequences^26^. A European study found medical students lacked consistent training on smoking cessation methods, suggesting that this challenge is not unique to Saudi Arabia^27^. Continued integration of smoking-related content into medical curricula is essential to address these deficiencies.

Regarding usage patterns, the most common nicotine dosage used was 10 mg, consistent with international consumer trends where users often seek products that offer higher nicotine delivery^28^. The decision to use nicotine pouches was frequently influenced by peers and online content, which reflects findings from Western studies that highlight the growing influence of social media platforms in shaping attitudes toward nicotine products^29^. Marketing tactics, particularly influencer-driven campaigns, have amplified interest in these products among young adults^30^. These dynamics raise concerns about the normalization of nicotine use and emphasize the importance of monitoring how such products are introduced and promoted in youth-oriented digital spaces^31^.

Participants expressed a mix of interest and caution regarding nicotine pouches. While some noted potential health-related motivations for switching to pouches, others questioned the lack of long-term evidence and the potential for new forms of dependence^16^. Although nicotine pouches do not contain tobacco leaf and may deliver fewer toxicants compared to cigarettes, nicotine itself remains highly addictive and can contribute to cardiovascular and other health risks^32^.

A substantial portion of respondents supported integrating education about nicotine pouches into medical training. This view aligns with international recommendations encouraging healthcare providers to stay informed about all nicotine products in order to offer evidence-based guidance ^32^. Participants also emphasized the need for stronger regulatory oversight and expressed skepticism about the role of the tobacco industry in promoting alternative products. This suggests a growing awareness of the commercial interests involved and highlights the need for transparent policy-making that prioritizes public health.

### Limitations

This study has several limitations that should be considered when interpreting the findings. First, the use of a non-random, convenience sampling method introduces a high risk of selection bias. Students who had a particular interest in smoking, nicotine products, or related topics may have been more inclined to participate, especially through online platforms. As a result, findings such as the reported rate of smoking cessation among nicotine pouch users may reflect a self-selected and potentially more engaged subgroup rather than a representative sample. This limits the generalizability of the results to the wider population of Saudi medical students. Second, the recruitment of participants from the selected universities may restrict the external validity of the study. These results may not apply to students in other regions, academic disciplines, or cultural settings. Third, the cross-sectional study design limits the ability to infer causal relationships between knowledge, perceptions, and nicotine pouch use. Fourth, the reliance on self-reported data introduces the potential for recall bias and social desirability bias, particularly regarding behaviors such as tobacco or nicotine use. Fifth, although multivariable regression analyses were used to adjust for potential confounding variables, unmeasured factors such as socioeconomic status, family influence, or prior health education may still have influenced the observed associations. Finally, because the study focused solely on medical students, the findings may not reflect the knowledge or behaviors of non-medical students or the general public. Medical students’ exposure to health-related curricula may shape their attitudes and awareness in ways that differ from other populations.

## CONCLUSIONS

This study reports the prevalence of smoking, knowledge, perceptions, and usage patterns of nicotine pouches among Saudi medical students, a key demographic for shaping future public health strategies. With a smoking prevalence of 16.3%, the findings underscore the ongoing challenge of tobacco use within this population. Notably, 58.6% of participants demonstrated good knowledge of nicotine pouches, with variations influenced by demographic and academic factors such as gender, academic year, and GPA. Concerns about nicotine dependence, regulatory oversight, and the normalization of smoking behaviors remain critical issues that warrant further attention. These results emphasize the importance of targeted, evidence-based educational interventions to enhance awareness and promote the responsible use of nicotine pouches among future healthcare professionals. Future research should explore the long-term effects of nicotine pouch use and examine broader population dynamics.

## Supplementary Material



## Data Availability

The data supporting this research are available from the author on reasonable request.
