# Supplementary material for: Knowledge, perceptions, and usage patterns of nicotine pouches among Saudi medical students: A cross-sectional study
Source: Tob Induc Dis. 2025 Sep 8;23:10.18332/tid/207914. doi: 10.18332/tid/207914 (PMC12413771; doi:10.18332/tid/207914)
Supplement: Supplementary file 1 [file TID-23-125_s1.pdf]

Figure 1. Distribution of nicotine dosage preferences (3 mg, 6 mg, 10 mg) among nicotine pouch users (n = 35), cross-sectional study, Saudi Arabia, 2024.

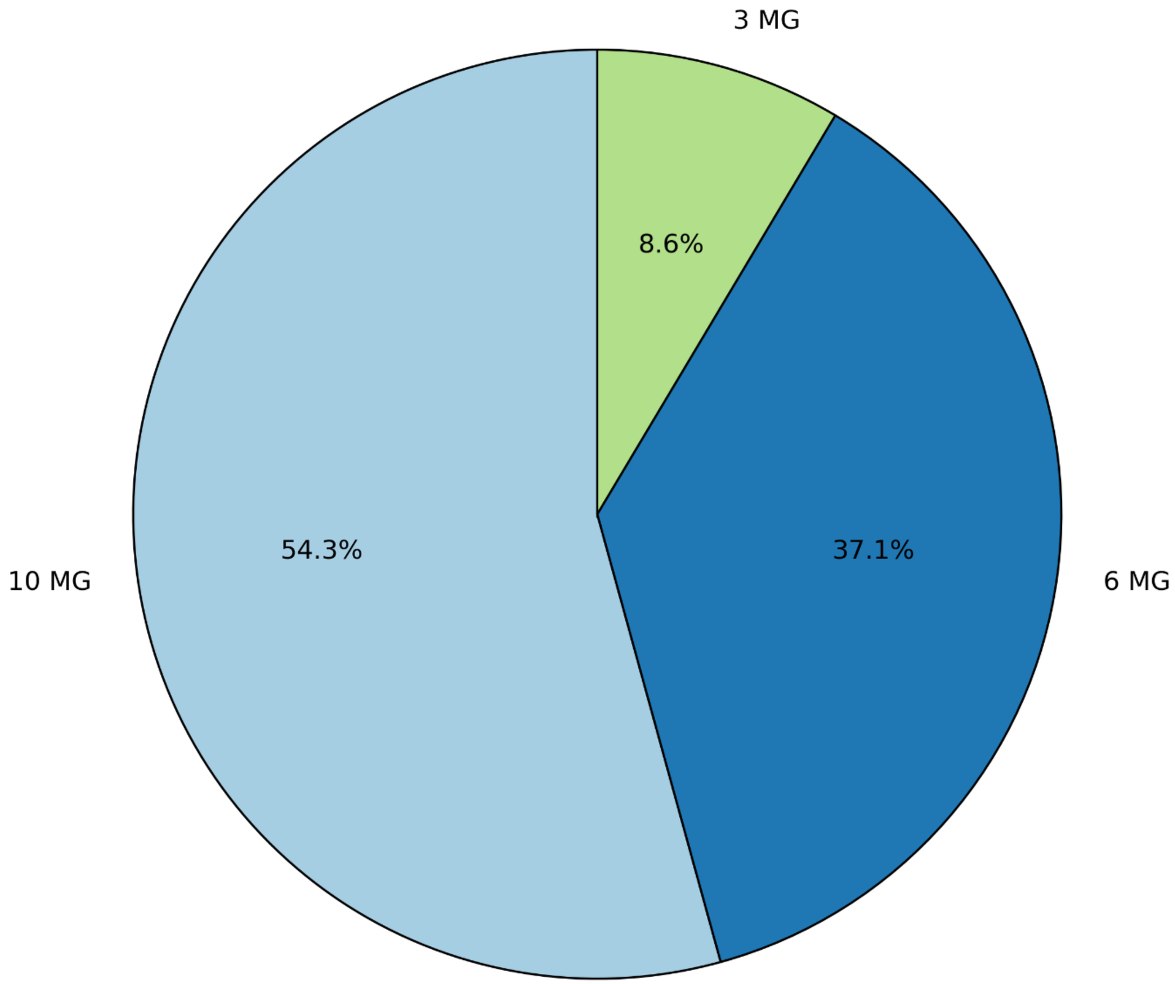

Figure 2. Changes in nicotine dosage over time among nicotine pouch users (n = 35), cross-sectional study, Saudi Arabia, 2024.

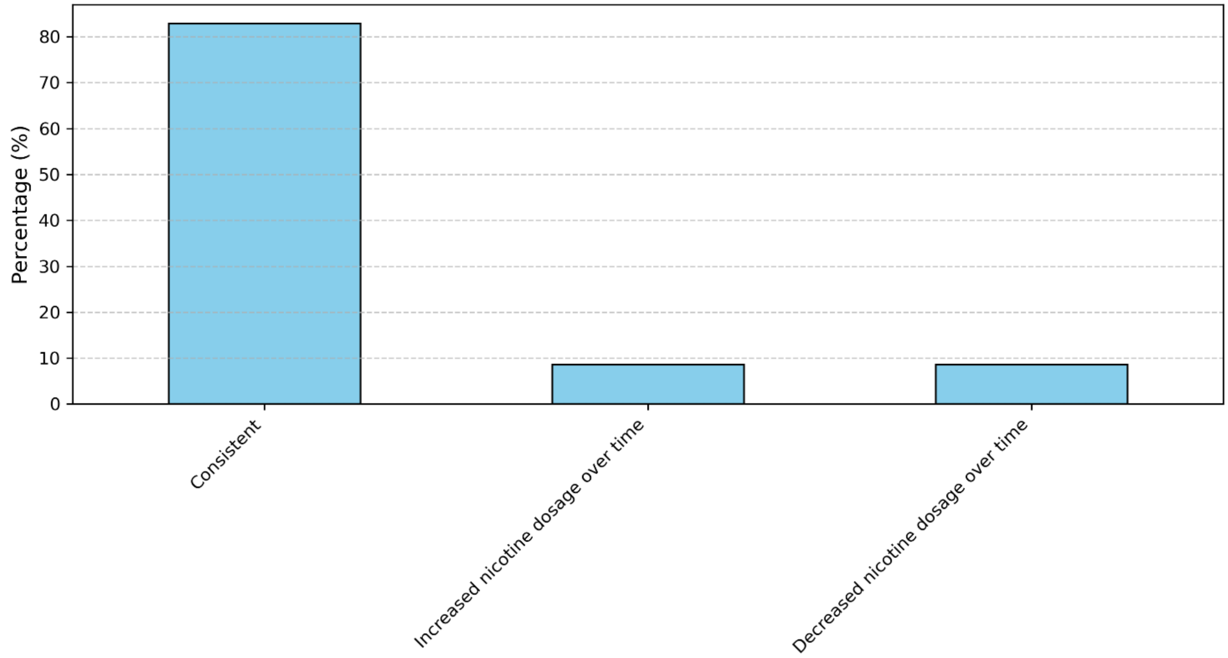

Figure 3. Factors influencing adoption of nicotine pouches among Saudi medical students who reported usage (n = 35), cross-sectional study, Saudi Arabia, 2024.

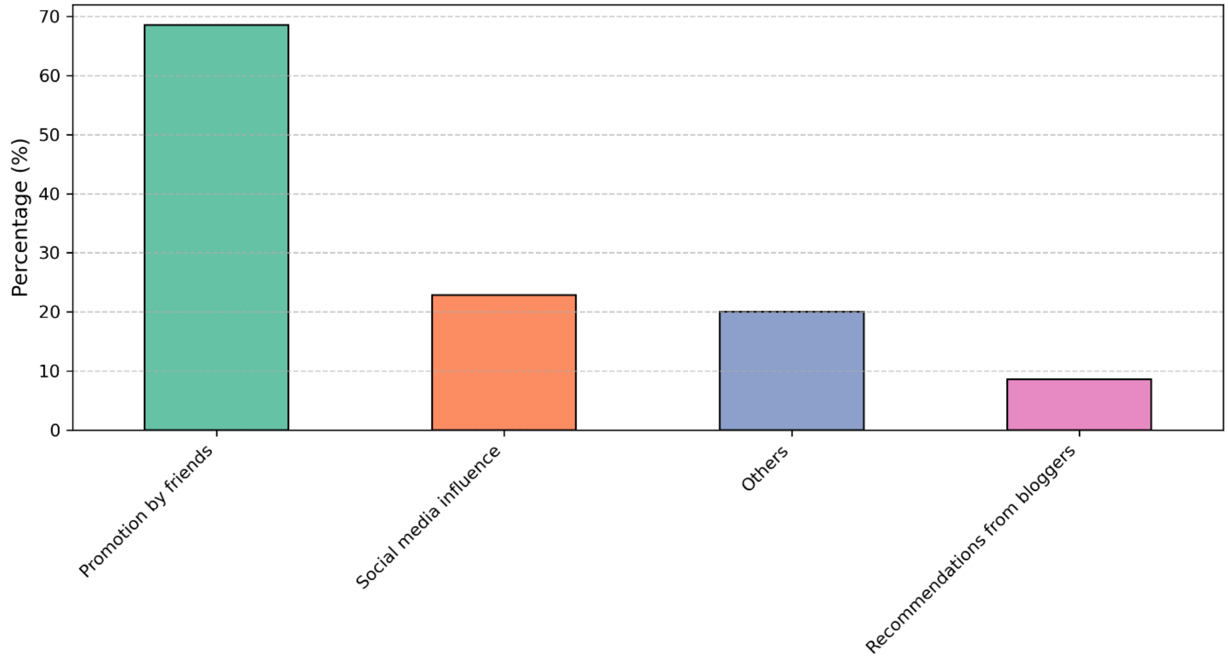

Figure 4. Sankey diagram illustrating the reported impact of nicotine pouch use on smoking behavior and perceived health outcomes among users (n = 35), cross-sectional study, Saudi Arabia, 2024.

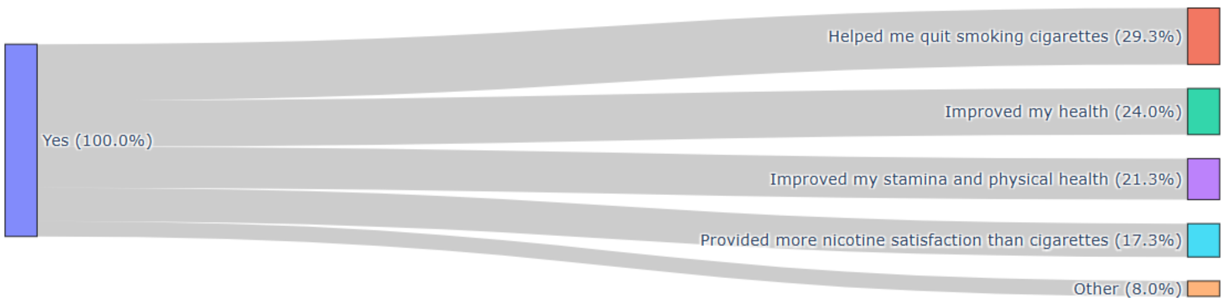

© 2025 Alshahrani N.Z.
